# Augmented Degradation of Factors VIII and IX in the Intermittent Movement State

**DOI:** 10.3390/ijms241310731

**Published:** 2023-06-27

**Authors:** Haim Cohen, Anat Keren-Politansky, Yonatan Crispel, Chen Yanovich, Keren Asayag, Yona Nadir

**Affiliations:** 1 Thrombosis and Hemostasis Unit, Department of Hematology, Rambam Health Care Campus, Haifa P.O. Box 9602, Israely_crispel@rambam.health.gov.il (Y.C.); hionobich@gmail.com (C.Y.);; 2 The Rappaport Faculty of Medicine, Technion, Haifa P.O. Box 9602, Israel

**Keywords:** factor VIII, factor IX, thrombin, movement

## Abstract

The most common clinical presentation of hemophilia A and hemophilia B is bleeding in large joints and striated muscles. It is unclear why bleeding has a predilection to affect joints and muscles. As muscles and joints are involved in intermittent movement, we explored whether this phenomenon could be associated with an impact on factor VIII and IX levels. Purified proteins and a mouse model were assessed using coagulation assays, Western blot analysis and immuno-staining. Movement caused an increase in thrombin activity and a decrease in factor VIII and factor IX activity. The decrease in factor VIII activity was more significant in the presence of thrombin and during movement. Under movement condition, sodium ions appeared to enhance the activity of thrombin that resulted in decreased factor VIII activity. Unlike factor VIII, the reduction in factor IX levels in the movement condition was thrombin-independent. High factor VIII levels were found to protect factor IX from degradation and vice versa. In mice that were in movement, factor VIII and IX levels decreased in the microcirculation of the muscle tissue compared with other tissues and to the muscle tissue at rest. Movement had no effect on von Willebrand factor levels. Movement induces reduction in factor VIII and IX levels. It enables an increase in the binding of sodium ions to thrombin leading to enhanced thrombin activity and augmented degradation of factor VIII. These data suggest a potential mechanism underlying the tendency of hemophilia patients to bleed in muscles and joints.

## 1. Introduction

Hemophilia A and B have similar clinical signs and symptoms [1]. In severe hemophilia, most bleeding events occur spontaneously in large joints and in muscles. Repeated bleeding in joints causes arthropathy, which is the major chronic complication of hemophilia. The handicap is often aggravated by muscle atrophy related to muscle bleedings [2]. Studies using preparations enriched in activated factor VIII have identified it as a cofactor of activated factor IX in the factor X-activating complex of the intrinsic coagulation pathway [3]. Factor VIII is synthesized as a 2351 amino acid single-chain glycoprotein. The domain organization is typically characterized as A1-A2-B-A3-C1-C2. The mature protein, with a molecular weight of 280 kDa, is composed of a light chain and a heavy chain. The light chain, having a molecular weight of 80 kDa, is made up of domains A3-C1-C2 [4,5]. Upon activation by thrombin, factor VIII is released from von Willebrand factor (vWF) and a series of specific proteolytic degradations occurs. Thrombin cleaves after arginine (Arg) residues 372, 740, and 1689. Cleavage after Arg-1689 is required to dissociate factor VIII from vWF. Cleavage after Arg-740 releases the dispensable B chain domain, while cleavage after Arg-372 allows the A1 domain to separate from the A2 domain, subsequently leading to complete dissociation of the activated complex. However, prior to its dissociation, factor VIII accelerates the rate of factor X activation by factor IXa and eventually contributes to the formation of a blood clot. Thus, thrombin has a dual effect: it activates factor VIII and then induces degradation of the latter. Likewise, activated protein C (APC) can cleave factor VIII at Arg-372 and induce its degradation. In addition, APC is capable of inactivating factor VIII by binding to residues 2009–2019 and cleaving in the A3 domain after factor VIII separation from vWF [4,5].

Thrombin possesses vital features, ensuring its specificity. These include loops and charged patches that surround the active site pocket. One of the essential loops comprises a Na^+^ binding site, whose occupancy regulates the interaction with other proteins. Na^+^ bound to thrombin, previously defined as the “fast” form in contrast to the unoccupied “slow” form [6], changes the enzyme function through expanding the access of small substrates to the active site and thus promoting certain procoagulant (e.g., with fibrinogen, PAR-1) and not anticoagulant interactions. The residues in the thrombin molecule that link to Na^+^ are the amino acids 189, 217, 222, and 225 [7]. Mutations in amino acid 217 [7,8] are capable of acquiring the conformation of the slow form of thrombin that may lead to marked attenuation in its procoagulant activity. To that end, such mutants could potentially be employed as anticoagulant therapeutic agents [9].

The purpose of the current research was to explore the predilection of hemophilia patients to bleed mainly in striated muscles and joints. Since, unlike other body tissues which are at steady rest or perpetual movement, both muscles and joints represent tissues that are in intermittent movement. We have hypothesized that this condition has an impact on the function or degradation of clotting factors VIII and IX.

## 2. Results

### 2.1. Effects of Movement on Purified Proteins

**Figure 1:**
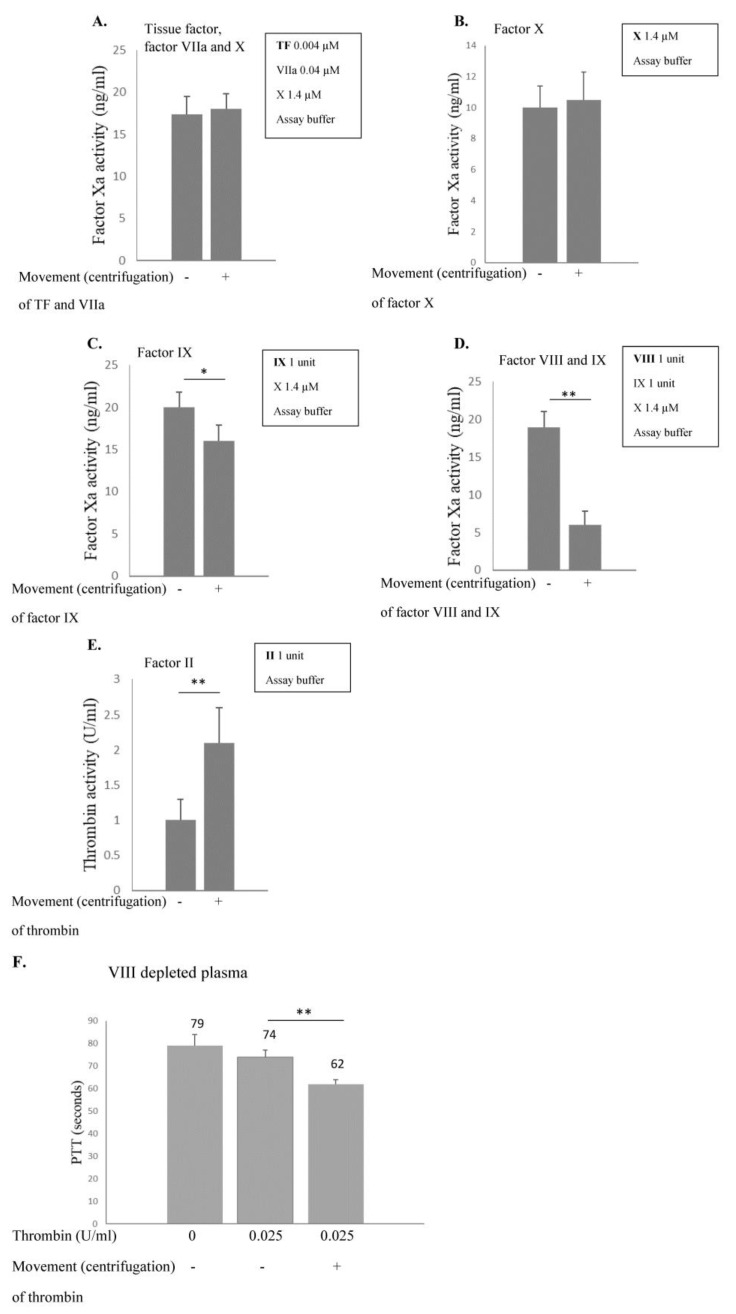
**Effects of movement on purified proteins.** Proteins were studied (**A**–**E**) at the indicated dose in the assay buffer (0.06 M Tris, 0.04 M NaCl, 2 mM CaCl_2_, 0.06 M BSA). The final assay volume was 2 mL in 15 mL tubes. Movement was produced by centrifugation at a rate of 100× *g* for 10 min. All coagulation factors, except for factor VIIa were plasma-derived that harbored traces of thrombin. Centrifugation increased activity of thrombin (**E**) and reduced that of factors VIII (**D**) and IX (**C**). Similar results were obtained when other means of movement were applied: 360° vertical circle rotator at a rate of 20 r/min. and a horizontal agitator at a rate of 30 r/min. Chromogenic substrate to factor Xa or thrombin (as indicated) was added (1 mM). Following 20 min, the reaction was stopped with 50 μL of glacial acetic acid and the activity of Xa or thrombin was determined using an ELISA plate reader (Power Wave XS, BIO-TEK, Vermont, USA). Bovine factor Xa or human thrombin diluted in the assay buffer was used to generate a standard curve. (**F**) Effect of movement on thrombin was further analyzed. Thrombin was added to the plasma deficient in factor VIII (1%) and the activity of the former was determined by the partial thromboplastin time (PTT) assay. When thrombin was centrifuged prior to addition to the factor VIII deficient plasma (100× *g* for 10 min), the effect of PTT shortening was more prominent. Factor VIII deficient plasma is known to have a prolonged PTT enabling more clear demonstration of the thrombin effect. Results represent mean of triplicates ±SD. * *p* < 0.05, ** *p* < 0.005.

In vitro movement was evaluated using purified proteins in the assay buffer [0.06 M Tris, 0.04 M NaCl, 2 mM CaCl_2_, 0.06 M BSA (bovine serum albumin), osmolarity 320]. The final assay volume of 2 ml was added to 15 mL tubes to enable effective movement that was produced by centrifugation at 100× *g* for 10 min. All coagulation factors, except for factor VIIa were derived from plasma that harbored traces of thrombin. Centrifugation followed by homogenization was found to increase the activity of thrombin (Figure 1E) as well as reduce that of factors VIII (Figure 1D) and IX (Figure 1C). Similar results were obtained when other means of movement were applied: a 360° vertical circle rotator at a rate of 20 r/min. and a horizontal agitator at a rate of 30 r/min. Subsequently, thrombin was added to plasma deficient in factor VIII (1%) and the activity of the former was determined by the PTT assay performed in 37 °C. When thrombin was centrifuged and homogenized prior to addition to factor VIII deficient plasma, the effect of PTT shortening was more prominent. We used factor VIII deficient plasma as it has a prolonged PTT enabling more clear demonstration of the thrombin effect (Figure 1F).

### 2.2. In the Presence of Thrombin, Factor VIII Levels Are Reduced during Movement

**Figure 2:**
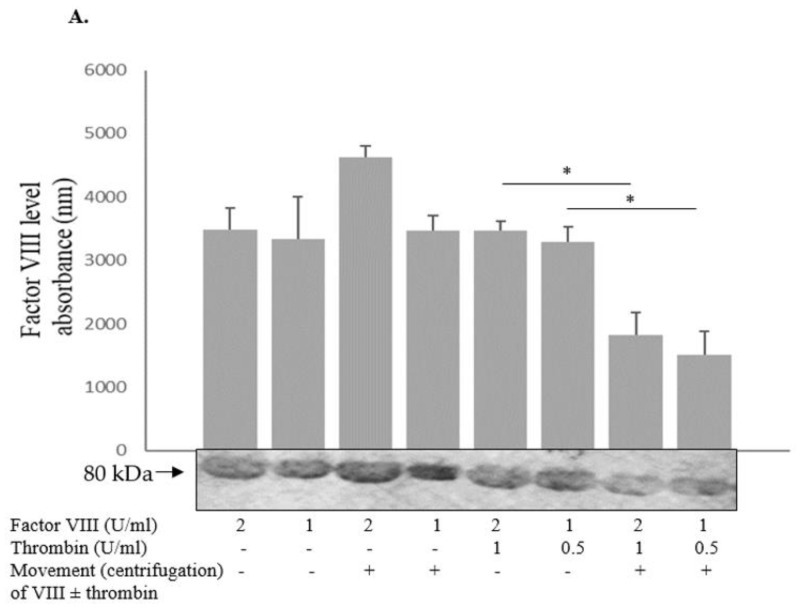

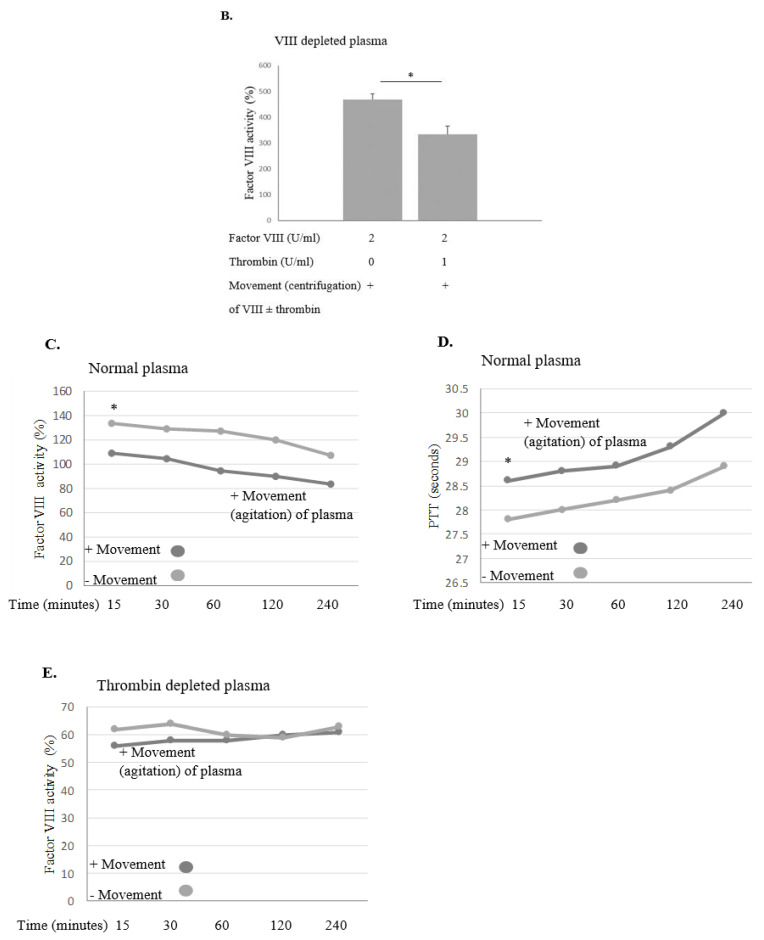

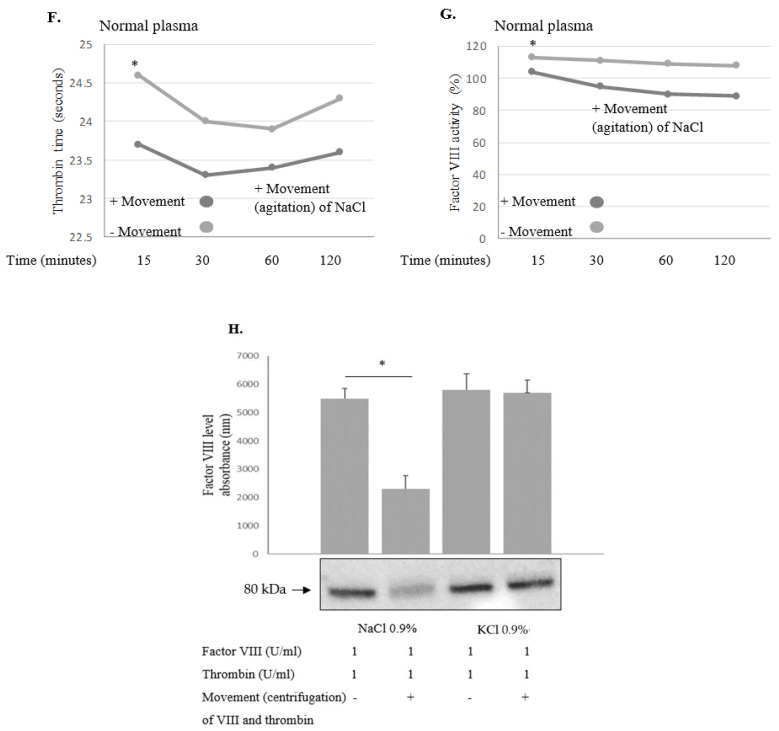

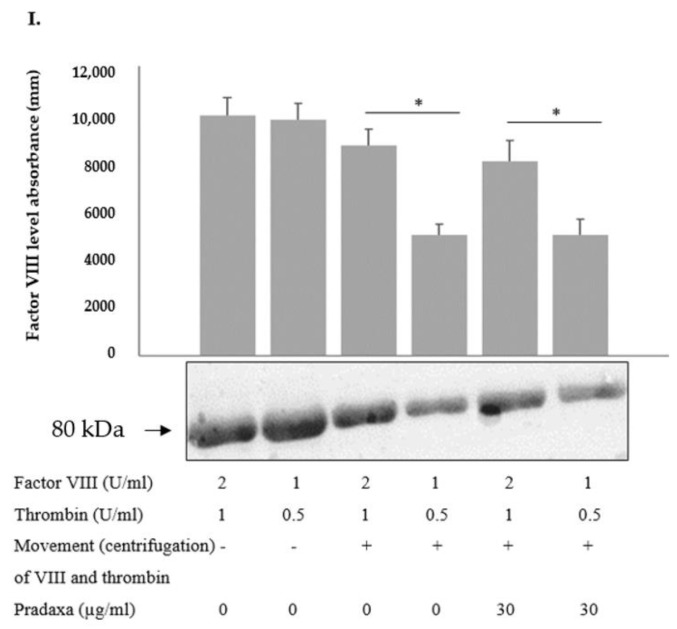
**Sodium ions mediate decreased factor VIII levels during movement in the presence of thrombin.** (**A**) Factor VIII with or without added thrombin was placed in a centrifuge (500× *g*) for 10 min at room temperature in the assay buffer (0.06 M Tris, 0.04 M NaCl, 2 mM CaCl_2_, 0.06 M BSA) and then the level of factor VIII was measured with Western blot (**A**). (**B**) A similar study was performed using factor VIII deficient plasma and evaluating factor VIII activity (Result represent mean of triplicates ±SD, * *p* < 0.05). Thrombin and movement decreased the level of factor VIII (**A**,**B**). Effects of horizontal movement in the agitator versus no-movement condition over 15, 30, 60, 120, 240 min (as indicated) were evaluated in normal plasma. No additional effect of prolonged movement time was observed (**C**,**D**). When thrombin-depleted plasma was evaluated for the indicated time length, no difference in factor VIII was observed over time (**E**), implying that the effect was mediated by thrombin. Effects of sodium solution (NaCl 0.9%) were evaluated in normal plasma after horizontal movement in the agitator compared to the no-movement condition over 15, 30, 60, 120 min (as indicated) following thrombin addition to the NaCl solution. Shaken NaCl induced shortening of thrombin time and reduced factor VIII activity (**F**,**G**). No effect of sodium solution shaking duration was observed. Results (**C**–**G**) represent mean of triplicates. Results (**C**–**G**) represent mean of triplicates. * *p* < 0.05. Significance was determined by Mann–Whitney U test. No statistically significant change was observed over time. (**H**) Factor VIII with thrombin were placed in a centrifuge (500× *g*) for 10 min at room temperature in 0.9% NaCl or 0.9% KCl. and then the level of factor VIII was measured with Western blot. Movement reduced factor VIII level in NaCl solution but not in KCl solution, indicating that Na^+^ ions were involved in the effect and not Cl^−^ or K^+^ ions. (**I**) Factor VIII with added thrombin was placed in a centrifuge (500× *g*) for 10 min at room temperature in the assay buffer (0.06 M Tris, 0.04 M NaCl, 2 mM CaCl_2_, 0.06 M BSA) and then the activity of factor VIII was assessed with Western blot. The thrombin inhibitor Pradaxa had no effect on preventing factor VIII degradation, implying that the inhibition site of thrombin by dabigatran is not involved in factor VIII degradation. Relative protein levels in Western blots were quantified by densitometry analysis (upper panel). Assays were performed in triplicate. The results represent mean ±SD. * *p* < 0.05. Significance was determined by Mann–Whitney U test.

Factor VIII with or without added thrombin was placed in a centrifuge (500× *g*) for 10 min at room temperature in the assay buffer (0.06 M Tris, 0.04 M NaCl, 2 mM CaCl_2_, 0.06 M BSA) and then the level of factor VIII was measured with Western blot (Figure 2A). A similar study was performed to evaluate factor VIII activity in plasma deficient in factor VIII at 37 °C (Figure 2B). Thrombin was found to decrease the levels of factor VIII under movement condition (Figure 2A,B). We subsequently evaluated if the effect of thrombin and movement on factor VIII activity changed over time. Endogenic factor VIII and thrombin in normal plasma were used to assess the effects of horizontal movement in the agitator versus no-movement condition over 15, 30, 60, 120, and 240 min. No additional effect of prolonged movement time was observed (Figure 2C,D), which could be related to a fixed dose of thrombin in the plasma sample anticoagulated with citrate. When thrombin-depleted plasma was evaluated for the indicated time period, no difference in factor VIII parameters was observed over time (Figure 2E), implying that the effect could be mediated by thrombin and the movement enhanced its activity.

### 2.3. Thrombin-Associated Decrease in Factor VIII Levels Is Mediated by Sodium Salt

As described in the Introduction Section, thrombin possesses features responsible for its specificity. One of the loops in the thrombin active site includes a Na^+^ binding site, which is involved in interaction with other proteins. We hypothesized that the ability of Na^+^ ions to bind to the thrombin active site could be enhanced by kinetic energy, i.e., movement. Effects of sodium solution (NaCl 0.9%) were evaluated in normal plasma after horizontal movement in the agitator compared to the no-movement condition for 15, 30, 60, and 120 min following thrombin addition (1 U/mL). Shaken NaCl 0.9% induced shortening of thrombin time and reduced factor VIII activity at 37 °C (Figure 2F,G), indicating that the movement of Na^+^ enhances thrombin activity in factor VIII degradation. No effect of shaking duration was observed. Factor VIII with thrombin were placed in a centrifuge (500× *g*) for 10 min at room temperature in 0.9% NaCl or 0.9% KCl. and then the level of factor VIII was measured with Western blot. Movement reduced factor VIII level in NaCl solution but not in KCl solution, indicating that Na^+^ ions were involved in thrombin activity enhancement and not Cl^−^ or K^+^ ions (Figure 2H).

### 2.4. Inhibition of Thrombin by Pradaxa (Dabigatran), When Added at Therapeutic Equivalent Concentration, Has No Effect on Factor VIII Degradation

Factor VIII with added thrombin was placed in a centrifuge (500× *g*) for 10 min at room temperature in the assay buffer (0.06 M Tris, 0.04 M NaCl, 2 mM CaCl_2_, 0.06 M BSA) and then the activity of factor VIII was assessed with Western blot. The thrombin inhibitor Pradaxa had no effect on preventing factor VIII degradation, implying that the inhibition active site of thrombin by dabigatran is not involved in the active site of factor VIII degradation (Figure 2I).

### 2.5. Factor VIII Levels in the Microcirculation of Mouse Striated Muscles Are Reduced following Movement

**Figure 3:**
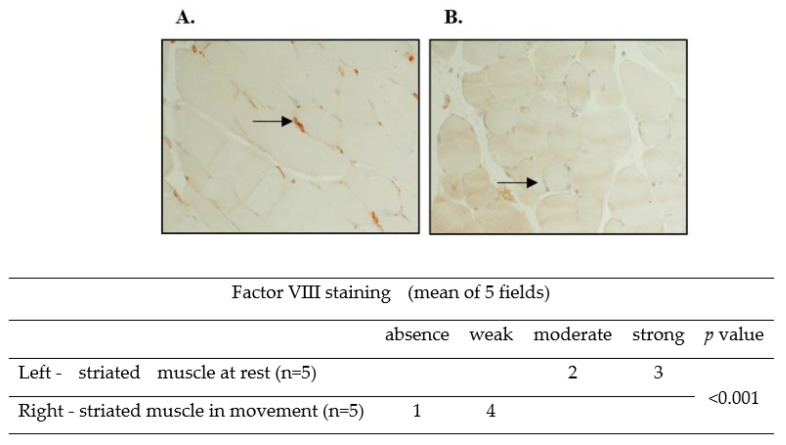

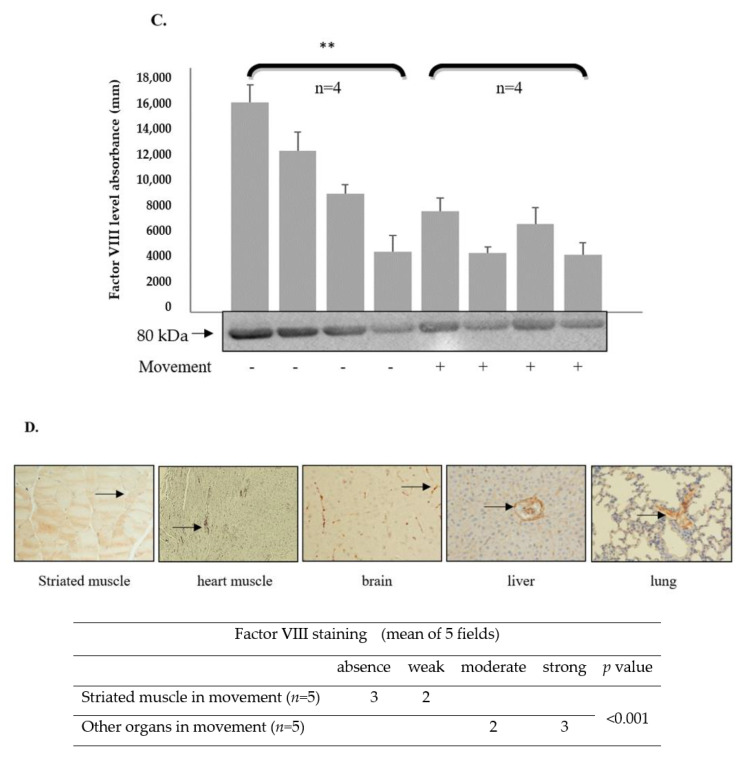
**Factor VIII levels in the microcirculation of mouse striated muscles are reduced following movement.** ICR mice (no specific genetic background) were put to sleep following isoflurane anesthesia (1.5%) for 15 min. Saturation was followed by pulse oximeter and temperature was kept by a heating lamp and monitored by a rectal thermometer. The study group (*n* = 5) continued regular activity in the cage. Both groups were sacrificed using isoflurane and CO_2_. The striated muscles of the legs were analyzed using immunostaining. A significant reduction in factor VIII levels in the microcirculation was observed in mice during movement compared to resting mice (black arrows, (**A**,**B**)). Representative images, captured with a Nikon E995 digital camera (Nikon, Tokyo, Japan). Original magnification, ×10. Table shows staining intensity in striated muscles. Significance was determined by U-test. (**C**) Lysate of muscles obtained either during movement or in the no-movement condition was subjected to Western blot analysis. Movement significantly reduced the level of factor VIII found in the muscle blood vessels. Relative protein levels in Western blots were quantified by densitometry analysis (upper panel). Assays were performed in triplicate. The results represent mean ± SD. ** *p* < 0.005. Significance was determined by Mann–Whitney U test. (**D**) The factor VIII level in the microcirculation of various organs, including heart muscle, brain, liver, and lung (black arrows) during movement was higher than that in striated muscles (magnification ×10). Representative images were captured with a Nikon E995 digital camera (Nikon, Tokyo, Japan). Table shows staining intensity in striated muscles and other organs.

At this stage, our hypothesis was tested in a mouse model. ICR mice (no specific genetic background, *n* = 5, control group) were put to sleep following isoflurane anesthesia (1.5%) for 15 min. Saturation was followed by pulse oximeter and temperature was kept by a heating lamp and monitored by a rectal thermometer. The study group (*n* = 5) continued regular activity in the cage. Both groups were sacrificed using isoflurane and CO_2_. The striated muscles of the legs were analyzed using immunostaining. A significant reduction in factor VIII levels in the microcirculation was observed in mice during movement compared to resting animals (Figure 3A,B), as indicated by immunostaining. Results were verified with Western blot analysis of muscle lysate (Figure 3C).

### 2.6. Factor VIII Levels in the Microcirculation of Organs in the Movement Condition

Comparison of factor VIII levels in the microcirculation of various organs, demonstrated its higher amounts in the heart muscle, brain, liver, and lung than those observed in the striated muscle during movement (Figure 3D).

### 2.7. High Levels of Factor IX Protect Factor VIII from Degradation

**Figure 4:**
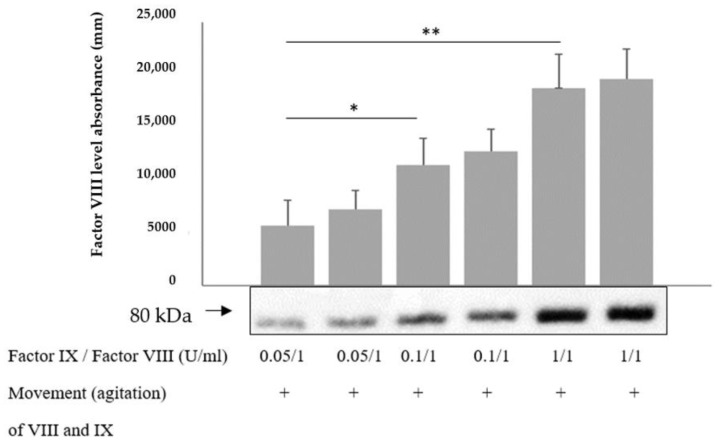
**High levels of factor IX protect factor VIII from degradation.** Factor VIII with added factor IX was placed in the horizontal agitator for 30 min at room temperature in the assay buffer (0.06 M Tris, 0.04 M NaCl, 2 mM CaCl_2_, 0.06 M BSA) and then the level of factor VIII was measured with Western blot. During movement, the level of factor VIII (at the indicated concentration) was maintained higher in the presence of factor IX. This correlation was dose-dependent. Relative protein levels in Western blots were quantified by densitometry analysis (upper panel). Assays were performed in triplicate. The results represent mean ± SD. * *p* < 0.05, ** *p* < 0.005. Significance was determined by Mann–Whitney U test.

It is well established that vWF protects factor VIII from degradation. In order to elucidate whether factor IX has a similar effect, factor VIII with added factor IX was placed in the horizontal agitator and shaken for 30 min at room temperature in the assay buffer (0.06 M Tris, 0.04 M NaCl, 2 mM CaCl2, 0.06 M BSA); then the level of factor VIII was measured with Western blot. During movement, levels of factor VIII (at the indicated concentration) were maintained higher in the presence of factor IX, with this correlation being dose-dependent (Figure 4). This finding suggests that factor IX deficiency may affect the factor VIII level, particularly in muscles, where it is reduced under movement condition. Notably, verification using Western blot analysis showed that the antibody of factor VIII failed to recognize factor IX and that of factor IX did not detect factor VIII.

### 2.8. Factor IX Degradation Is Enhanced by Movement and Preserved in the Presence of Factor VIII

**Figure 5:**
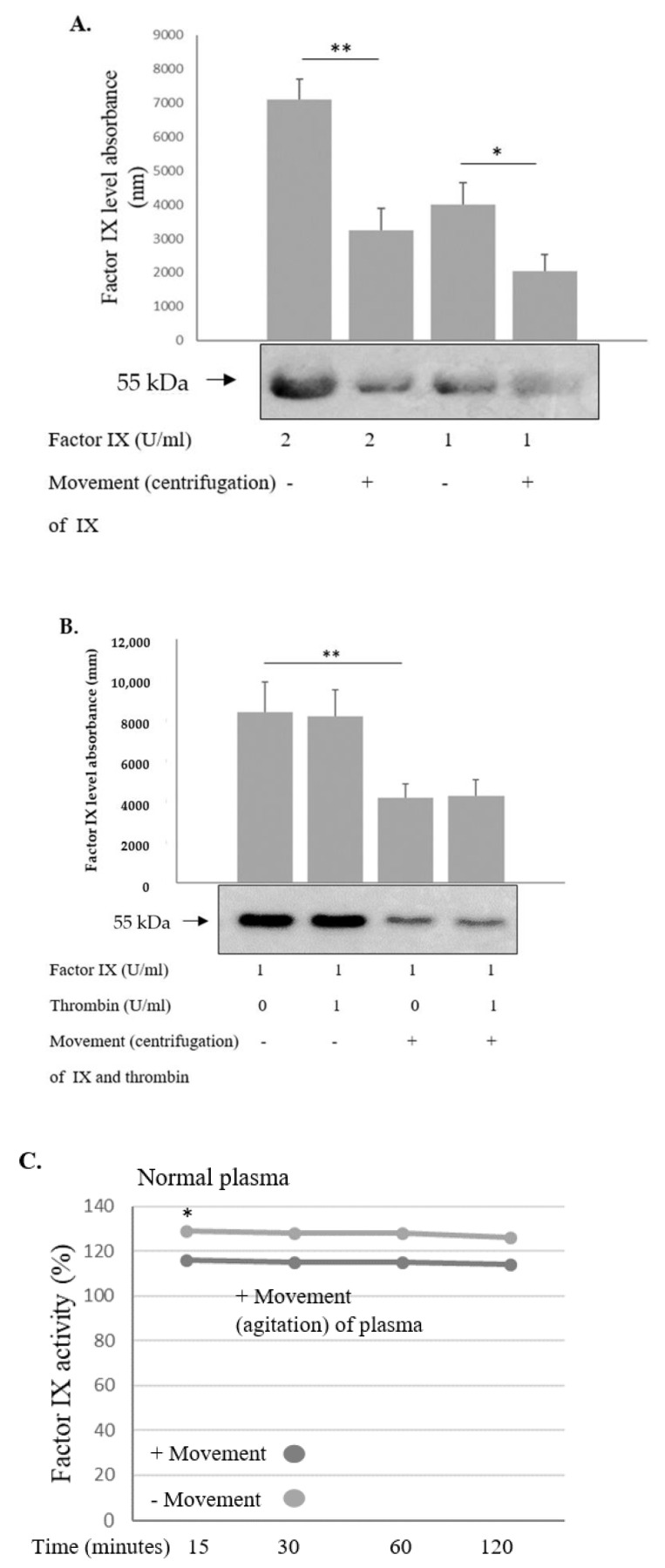

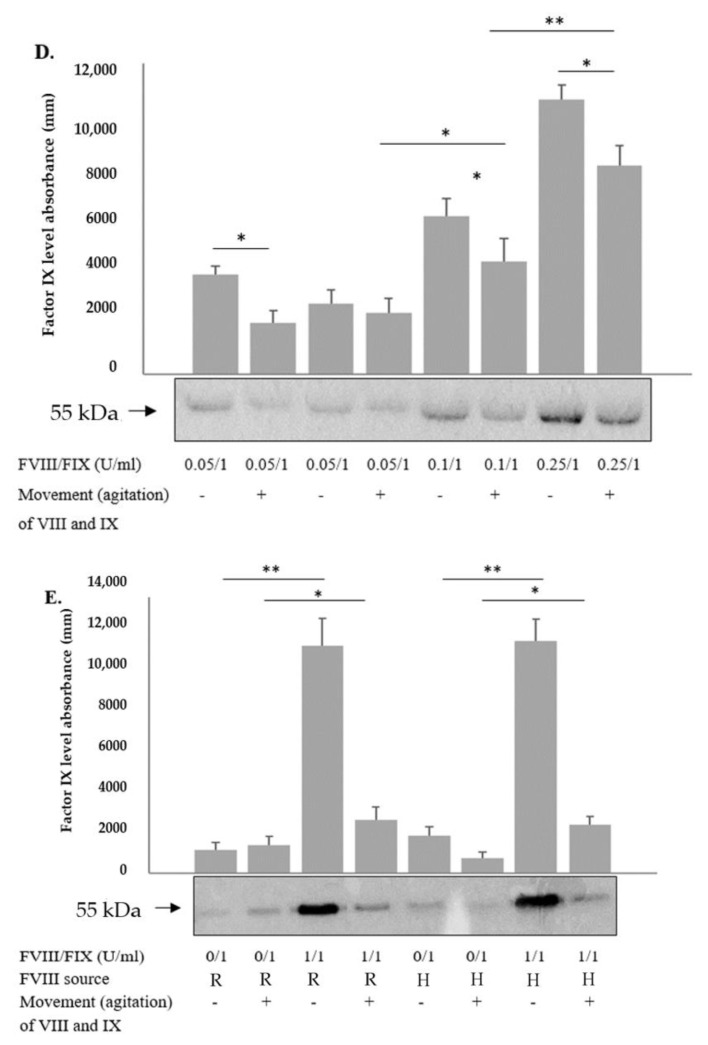
**Movement enhanced degradation of factor IX, which was protected in the presence of factor VIII.** (**A**) Factor IX was placed in a centrifuge (500× *g*) for 10 min at room temperature in the assay buffer (0.06 M Tris, 0.04 M NaCl, 2 mM CaCl_2_, 0.06 M BSA) and then its level was measured with Western blot. Movement enhanced degradation of factor IX in contrast to the no-movement condition. (**B**) The effect was not augmented in the presence of thrombin. (**C**) Effects of horizontal movement in the agitator versus no-movement condition over 15, 30, 60, 120 min (as indicated) were evaluated in normal plasma. No additional effect of prolonged movement time was observed. Results represent mean of triplicates. * *p* < 0.05. Significance was determined by Mann–Whitney U test. (**D**) When factor VIII was added to the assay, it prevented factor IX degradation in a dose-dependent manner. (**E**) The latter effect was observed using recombinant factor VIII (R) or human plasma derived (H) factor VIII. Relative protein levels in Western blots were quantified by densitometry analysis (upper panel). Assays were performed in triplicate. The results represent mean ± SD. * *p* < 0.05, ** *p* < 0.005. Significance was determined by Mann–Whitney U test.

The effect of movement on factor IX was investigated in the way similar to that of factor VIII. Factor IX was placed in a centrifuge (500× *g*) for 10 min at room temperature in the assay buffer (0.06 M Tris, 0.04 M NaCl, 2 mM CaCl_2_, 0.06 M BSA) and then its level was measured with Western blot. Movement was found to enhance degradation of factor IX compared to the no-movement condition (Figure 5A); however, unlike in the case of factor VIII, thrombin did not augment this effect (Figure 5B). When factor VIII was added to the assay, it prevented factor IX degradation in a dose-dependent manner (Figure 5C). The latter effect was observed using recombinant factor VIII or human plasma derived factor VIII (Figure 5D). These experiments were performed without thrombin addition, although a minimal level of thrombin is known to be present in factor IX.

### 2.9. Factor IX Levels in the Microcirculation of Mouse Striated Muscles Are Reduced following Movement

**Figure 6:**
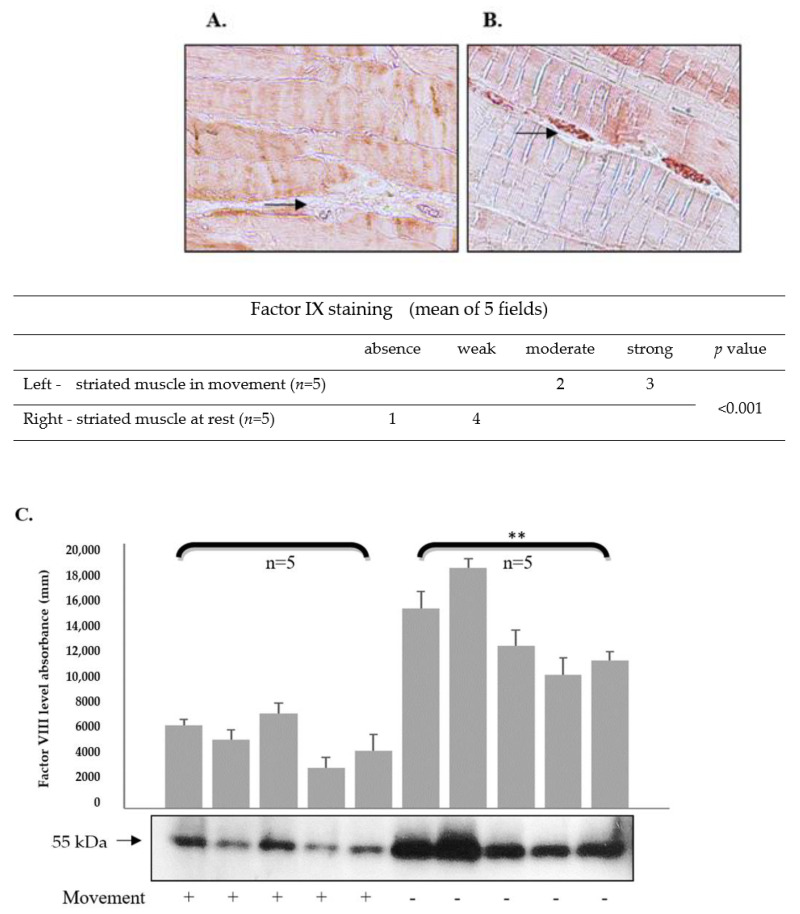
**Factor IX levels in the microcirculation of mouse striated muscles are decreased following movement.** ICR mice (no specific genetic background, *n* = 5, control group) were put to sleep following isoflurane anesthesia for 15 min. The study group (*n* = 5) continued regular activity in the cage. Both groups were sacrificed, and the striated muscles of the legs were assessed using immunostaining. A significant reduction in factor IX in the microcirculation was observed in mice during movement compared to resting mice (black arrows, (**A**,**B**)). Representative images, captured with a Nikon E995 digital camera (Nikon, Tokyo, Japan). Original magnification, ×10. The table shows staining intensity in striated muscles. Significance was determined by U-test. (**C**) Lysate of muscles obtained either during movement or in the no-movement condition was subjected to Western blot analysis. Movement significantly reduced the level of factor IX found in the muscle blood vessels. Relative protein levels in Western blots were quantified by densitometry analysis (upper panel). Assays were performed in triplicate. The results represent mean ± SD. ** *p* < 0.005. Significance was determined by Mann–Whitney U test.

ICR mice (no specific genetic background, *n* = 5, control group) were put to sleep following isoflurane anesthesia (1.5%) for 15 min. Saturation was followed by pulse oximeter and temperature was kept by a heating lamp and monitored by a rectal thermometer. The study group (*n* = 5) continued regular activity in the cage. Both groups were sacrificed using isoflurane and CO2. The striated muscles of the legs were analyzed. A significant reduction in factor IX levels in the microcirculation was observed in mice during movement compared to resting mice (Figure 6A,B). The muscle lysate obtained either during movement or in the no-movement condition was subjected to Western blot analysis. Movement was found to significantly reduce the level of factor IX found in the muscle blood vessels (Figure 6C).

### 2.10. Movement Does Not Affect the vWF Level

**Figure 7:**
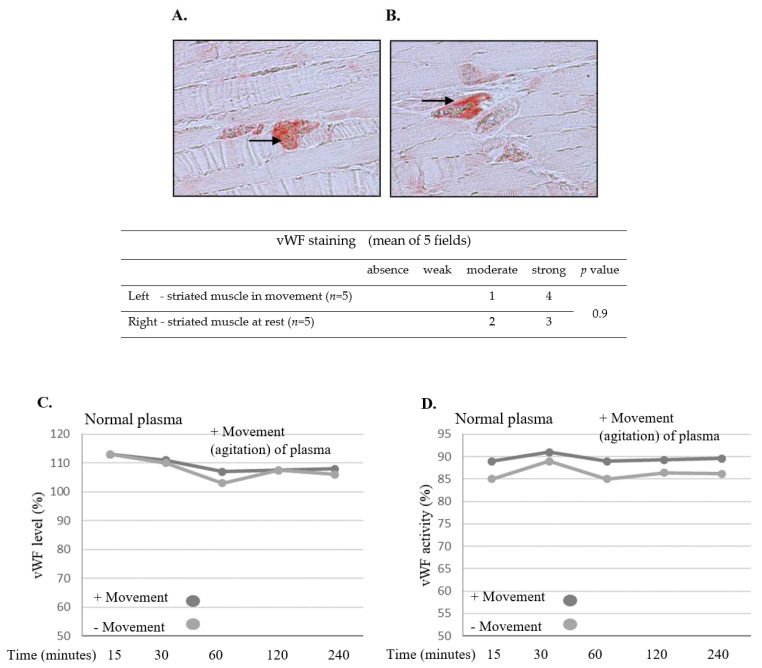
**No effect of movement on vWF level was observed.** ICR mice (no specific genetic background, *n* = 5, control group) were put to sleep following isoflurane anesthesia for 15 min. The study group (*n* = 5) continued regular activity in the cage. Both groups were sacrificed, and the striated muscles of the legs were analyzed using immunostaining. No effect on vWF microcirculation levels was observed in mice during movement compared to resting mice (black arrows, (**A**,**B**)). Representative images, captured with a Nikon E995 digital camera (Nikon, Tokyo, Japan). Original magnification, ×10. The table shows staining intensity in striated muscles. Significance was determined by U-test. Effects of horizontal movement in the agitator versus no-movement condition over 15, 30, 60, 120, and 240 min (as indicated) were evaluated in normal plasma. Movement did not decrease vWF levels and slightly increased its activity. No additional effect of prolonged movement time was observed (**C**,**D**). Results shown in (**C**,**D**) represent mean of triplicates.

ICR mice (no specific genetic background, *n* = 5, control group) were put to sleep following isoflurane anesthesia (1.5%) for 15 min. Saturation was followed by pulse oximeter and temperature was kept by a heating lamp and monitored by a rectal thermometer. The study group (n = 5) continued regular activity in the cage. Both groups were sacrificed using isoflurane and CO_2_. The striated muscles of the legs were investigated using immunostaining. No difference in the vWF microcirculation level was identified in mice during movement compared to resting animals (Figure 7A,B). The impact of exposure to horizontal movement in the agitator versus no-movement condition for 15, 30, 60, 120, and 240 min (as previously described) was evaluated in normal plasma. Movement did not decrease the amounts of vWF and slightly increased its activity. No additional effect of prolonged movement time was observed (Figure 7C,D).

## 3. Discussion

**Figure 8:**
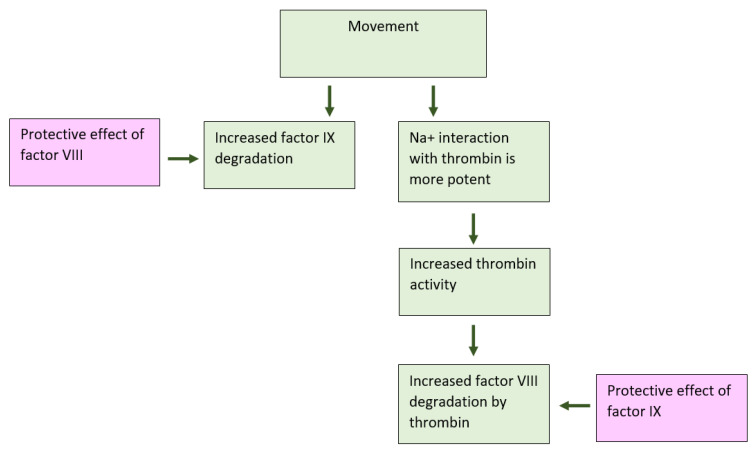
**Schematic summarization of intermittent movement effects on factor VIII and IX degradation.** Factor VIII and IX levels are decreased during movement. Factor IX protects factor VIII from degradation and vice versa. Thrombin activity is enhanced in movement due to an increased effect of Na^+^ ions in its active site. Augmented degradation of factor VIII is due to enhanced activation of thrombin.

The aim of the current research has been to explore the predilection of hemophilia patients to bleed mainly in striated muscles and joints. The phenomenon of spontaneous bleeding to muscles and joints in patients with severe hemophilia has raised the question regarding the cause of such predilection. The present study has demonstrated that thrombin activity is enhanced during movement (Figure 1). Thrombin is known to activate and degrade factor VIII [6]. We have found that factor VIII levels and activity are reduced during movement, which may be attributed to enhanced thrombin activity (Figure 2), given that in thrombin-deficient plasma, levels of factor VIII have remained unchanged (Figure 2). As to the mechanism underlying these findings, it has been previously reported that binding of Na^+^ ions to a thrombin-specific site is important for the interaction with other proteins [8]. We have observed that during movement, Na^+^ ions enable augmented thrombin activity (Figure 2). We assume that movement, which is kinetic energy, may turn into chemical energy, facilitating Na^+^ ion interaction with the thrombin active site. While we have not identified the exact site of thrombin interaction with factor VIII, it has been demonstrated that dabigatran, an inhibitor of thrombin that prevents the conversion of fibrinogen into fibrin, is not the site involved (Figure 2). Findings of our in vitro experiments have been verified in a mouse model, showing that factor VIII levels in the microcirculation of mouse striated muscles are decreased following movement (Figure 3), in contrast to other organs such as heart muscle, brain, liver and lung (Figure 3). Furthermore, factor IX has been found to protect factor VIII from degradation under movement condition (Figure 4). Actually, the factor VIII level has been maintained in the presence of factor IX at a ratio of 1:1, whereas lower levels of factor IX were associated with increased factor VIII degradation (Figure 4). Similar to factor VIII, movement has resulted in augmented degradation of factor IX. The effect has been independent of thrombin, although its traces are present in factor IX preparation. The finding of decreased factor IX amount in the microcirculation of mouse striated muscles following movement strengthens our results (Figure 5, Figure 6). Interestingly, factor VIII at a ratio of 1:1 has significantly prevented the degradation of factor IX, even under movement condition. The protective effect of factor VIII has been observed using human plasma derived or recombinant factor VIII (Figure 6). These findings may imply that normal levels of factor IX protect factor VIII from degradation and vice versa. Accordingly, a low level of one of these factors may reduce that of the other. This reciprocal effect may have biological significance in movement, especially if one of the factors is endogenously markedly reduced. No effect of movement on the level or activity of vWF in plasma or mouse model has been demonstrated (Figure 7). Summarized results are presented in Figure 8. showing that factor VIII and IX levels are decreased during movement. Thrombin activity is enhanced in movement due to an increased effect of Na^+^ ions in its active site and augmented degradation of factor VIII is due to enhanced activation of thrombin. Factor IX protects factor VIII from degradation and vice versa.

Although reduction in factors VIII and IX in striated muscles during movement may have no hemostatic effect in normal physiology, in patients with hemophilia it may explain a predilection to bleed in these muscles. Further investigation in organs such as the heart and bowels is warranted to explore why organs that are in constant movement do not display a decrease in the levels of factors VIII and IX. The impact of movement on these factors may have implications for blood banks in terms of preparation of plasma and cryoprecipitate. In recent years, there has been renewed enthusiasm for whole blood transfusion in the form of cold-stored low titer group O whole blood (LTOWB). Multiple studies in have documented its safety [10] and emerging data suggest promising outcomes, including mortality, reduced blood product requirements [11], and improved coagulation profile [12]. Rahbar et al. evaluated the levels of coagulation factors in the trauma patients’ plasma immediately following transfusion of whole blood (*n* = 15) compared to packed red blood cells and fresh-frozen plasma (FFP). All coagulation proteins were lower in the FFP group, although the decrease in factors IX, VIII, V, fibrinogen, and protein C was ≥14% and that in factors II, X, VII, and anti-thrombin was ≤10%. According to our results, the higher reduction in factor VIII and IX may be attributed to pre-centrifugation of the plasma prior to its use [12]. The protective role of factor IX on factor VIII, mediated by protein C, had been previously demonstrated [13]. The current study showed that the effect was reciprocal, i.e., factor VIII also protected factor IX from degradation. In addition, we demonstrated that the effect persisted under movement condition. This mechanism implies that in severe hemophilia, the decrease in factor VIII or IX levels is augmented by the lack of protective effect on the reciprocal factor.

It has been reported that following activation by thrombin, factor VIII loses its activity within minutes due to spontaneous dissociation of the A2-domain [14]. Conversely, earlier studies in purified systems demonstrate that inactivation of factor VIII by APC takes hours [15]. The higher rate of A2-domain dissociation relative to APC cleavage points to the former process as the primary mechanism underlying factor VIIIa inactivation [16]. This suggestion is supported by the fact that no clinical phenotype corresponding to altered APC cleavage of factor VIII has been reported. At the same time, in case of its homologous factor V, APC resistance (factor V Leiden, Arg506Gln) conveys a risk of venous thromboembolism that is increased by 50–100-fold and 5–10-fold in homozygous and heterozygous patients, respectively, and mutation in this factor is known to be associated with the most prevalent inherited thrombophilia [17].

While currently available data may put in doubt an impact of APC on the control of factor VIIIa activity, a recent study conducted by Wilhelm et al. demonstrated clinical relevance of inhibiting APC in a mouse model [18]. This is an elegant study of clinical relevance and the question whether the effect of factor VIII degradation by APC is enhanced during movement warrants further investigation.

Movement-related activation of thrombin is an intriguing finding. It was previously demonstrated that exhaustive exercise led to activation of blood coagulation in healthy subjects, including increased thrombin generation, shortened PTT and elevated levels of fibrin peptides 1 and 2. In a recent study by Ljungkvist et al., factor VIII was found to be vital for enhancing global coagulation after physical exercise. In the latter study, rotational thromboelastometry (ROTEM) and thrombin generation assay showed significantly augmented coagulation capacity after maximal exercise in healthy controls (*n* = 10) but not in patients with severe hemophilia A (*n* = 10). While vWF antigen and activity levels appeared to be significantly elevated in both groups, factor VIII levels demonstrated a significant increase in the control group only [19].

The current study has some drawbacks. The main limitation is that it gives no explanation why organs that are in constant movement, such as the heart and bowels, do not display a decrease in the levels of factors VIII and IX. Another restriction is that the effect was not demonstrated in hemophilic mouse models that could have strengthen the validity of the results. Even though, the findings of the present study point to a reduction in factor VIII and IX in intermittent movement and to a protective effect of factor IX on factor VIII degradation and vice versa. In addition, novel mechanism of activation of the coagulation system following intermittent movement was revealed. We demonstrated that thrombin activation is enhanced by Na^+^ ions, which interact more potently with factor VIII during movement. In view of factor VIII importance in exercise-induced hypercoagulability, identification of the specific site in thrombin involved in factor VIII activation may enable development of an anticoagulant drug specific to inhibition of factor VIII, without blocking the extrinsic pathway that is critical in injury, for patients who pursue an athletic lifestyle.

## 4. Materials and Methods

### 4.1. Reagents and Antibodies

Recombinant human factor VIIa, re-lipidated recombinant tissue factor (TF) and plasma-derived human factor X were purchased from Sekisui Diagnostics, Inc. (Stamford, CT, USA). Chromogenic substrate to factor Xa (I.D. 222, solubility: Tris buffer, pH 8.4) and chromogenic substrate to thrombin (I.D. 238, solubility: Tris buffer, pH 8.4) were purchased from Sekisui Diagnostics, Inc. Bovine factor Xa was obtained from Sigma (St. Louis, MO, USA) and human thrombin was purchased from Siemens (Munich, Germany). Plasma derived Factor VIII Hemofil-M was obtained from Baxter (Placer County, CA, USA). Recombinant Factor VIII XYNTHA was acquired from Pfizer (New York, NY, USA). Plasma derived Factor IX Replenine—VF was purchased from Bio Products Laboratory (Hertfordshire, UK). Factor VIII depleted plasma and thrombin depleted plasma were obtained from Instrumentation Laboratory (Milano, Italy). All coagulation factors were dissolved in double-distilled water. Dabigatran was purchased from Boehringer Ingelheim (Rheine, Germany) and Polyclonal anti factor VIII light chain (H-100) and polyclonal anti vWF were purchased from Santa Cruz Biotechnology, Inc. (Heidelberg, Germany). Polyclonal anti factor IX was obtained from Nevus Bioligicals Europe (Abingdon, UK).

### 4.2. Mouse Model

The study was approved by the Technion Ethics Committee for Animal Research and the procedures followed were in accordance with institutional guidelines. We studied ICR mice (no specific genetic background). All the experiments were performed in seven-to-eight-week-old male mice in order to avoid hormonal effects on factor VIII.

### 4.3. Coagulation Assays

The partial thromboplastin time (PTT) assay was performed on the Sysmex CA1500 analyzer (Siemens Healthcare Diagnostics, Marburg, Germany). Dade Actin FS Activated PTT Reagent was used for PTT (Siemens Healthcare Diagnostics). Levels of coagulation factor VIII activity were determined using a 1-stage assay with factor VIII deficient plasma (Diagnostica Stago) and determined on the Sysmex CA1500 analyzer (Siemens Healthcare Diagnostics, Marburg, Germany). The thrombin time (TT) assay was performed on the ACL-TOP550 coagulation system (Instrumentation Laboratory Company, Barcelona, Spain). Bovine thrombin reagent (Instrumentation Laboratory, Lexington, MA, USA) was added to plasma.

### 4.4. Factor Xa Chromogenic Assay

An amount of 50 μL of sample was added to 50 μL of Tris buffer (0.06 M, pH 8.4) and 25 μL of chromogenic substrate to factor Xa (5 mM). After 30 min, the reaction was stopped with glacial acetic and the absorbance at 405 nm and 490 nm was measured using an ELISA plate reader (Power Wave XS, BIO-TEK). Bovine factor Xa diluted in the assay buffer was used to generate a standard curve.

### 4.5. Thrombin Chromogenic Assay

An amount of 50 μL of a sample were added to 50 μL of Tris buffer (0.06 M, pH 8.4) and 25 μL of chromogenic substrate to thrombin (5 mM). After 30 min, the reaction was stopped with glacial acetic and the absorbance at 405 nm and 490 nm was measured using an ELISA plate reader (Power Wave XS, BIO-TEK). Human thrombin diluted in the assay buffer was used to generate a standard curve.

### 4.6. SDS-Polyacrylamide Gel Electrophoresis (PAGE) and Immunoblot Analysis

In each sample, 40 µg of protein was loaded to the gel. Proteins were subjected to 10% SDS-PAGE and transferred to the polyvinylidene fluoride membrane (BioRad, Maylands, CA, USA). The membrane was probed with the appropriate antibody followed by horseradish peroxidase-conjugated secondary antibody (Jackson ImmunoResearch, West Grove, PA, USA) and chemiluminescence substrate (Pierce, Rockford, IL, USA). Rabbit anti-factor VIII light chain, rabbit anti-factor IX, or rabbit anti-vWF diluted to 1/2000 was used.

### 4.7. Immunohistochemistry

Staining of formalin-fixed, paraffin-embedded 5-micron sections of muscles was performed. Slides were deparaffinized with xylene, rehydrated and endogenous peroxidase activity was quenched for 30 min by 3% hydrogen peroxide in methanol. Slides were then subjected to antigen retrieval by boiling (20 min) in 10 mM citrate buffer, pH 6. Slides were incubated with 10% normal goat serum in PBS for 60 min to block non-specific binding, which was followed by incubation (20 h, 4 °C) with anti-factor VIII, anti-factor IX, or anti-vWF (1:250 dilution). Slides were subsequently extensively washed with PBS containing 0.01% Triton X-100 and incubated with a secondary antibody (Envision kit; Dako, Glostrup, Denmark) according to the manufacturer’s instructions. Following additional washes, color was developed with the AEC reagent (Sigma, St. Louis, MO, USA) and sections were counterstained with hematoxylin and mounted. Analyses of tissues immunohistochemistry results were performed by two of the authors unaware of the slide allocation. Discrepancies in the analyses were reconciled following the assessment by a third reviewer. Five high power fields were evaluated in each stained slide. Staining intensity was scored as follows: 0, no staining; 1, weak intensity; 2, moderate intensity; and 3, marked intensity.

### 4.8. Means of Movement

Centrifugal, vertical by laboratory rotator or horizontal by platelets agitator (Please refer to Appendix A).

### 4.9. Statistical Analysis

Data were evaluated using SPSS software for Windows version 13.0 (SPSS Inc., Chicago, IL, USA). Statistics were calculated using the non-parametric Mann–Whitney-U test. Values were reported as median and range or mean ± SD, as indicated. The significance level was set at *p* < 0.05.

## Figures and Tables

**Figure A1:**
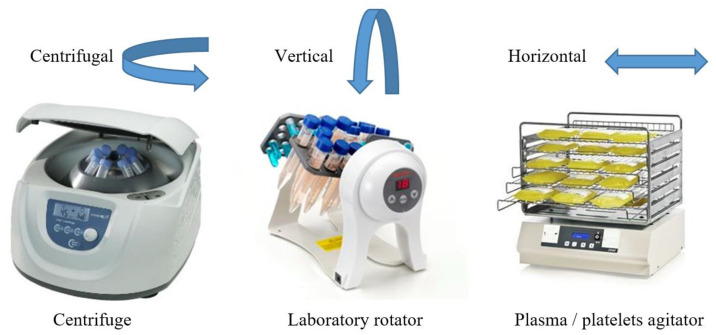
Centrifugal, vertical by laboratory rotator or horizontal by platelets agitator.
